# Biotics (Pre-, Pro-, Post-) and Uremic Toxicity: Implications, Mechanisms, and Possible Therapies

**DOI:** 10.3390/toxins15090548

**Published:** 2023-09-04

**Authors:** Laura Mitrea, Mădălina Medeleanu, Carmen-Rodica Pop, Ancuța-Mihaela Rotar, Dan-Cristian Vodnar

**Affiliations:** 1 Faculty of Food Science and Technology, University of Agricultural Sciences and Veterinary Medicine, 400372 Cluj-Napoca, Romania; laura.mitrea@usamvcluj.ro (L.M.); madalina.medeleanu@usamvcluj.ro (M.M.); anca.rotar@usamvcluj.ro (A.-M.R.); 2 Life Science Institute, University of Agricultural Sciences and Veterinary Medicine, 400372 Cluj-Napoca, Romania

**Keywords:** gut microbiota, dysbiosis, uremic toxins, kidney disorders, prebiotics, probiotics, postbiotics, therapies

## Abstract

In recent years, more scientific data have pointed out the close connection between intestinal microbial community, nutritional habits, lifestyle, and the appearance of various affections located at certain anatomical systems. Gut dysbiosis enhances the formation and accumulation of specific metabolites with toxic potential that induce the appearance of kidney-associated illnesses. Intestinal microbes are involved in the degradation of food, drugs, or other ingested products that lead to the formation of various metabolites that end up in renal tissue. Over the last few years, the possibilities of modulating the gut microbiota for the biosynthesis of targeted compounds with bioactive properties for reducing the risk of chronic illness development were investigated. In this regard, the present narrative review provides an overview of the scientific literature across the last decade considering the relationship between bioactive compounds, pre-, pro-, and post-biotics, uremic toxicity, and kidney-associated affections, and the possibility of alleviating the accumulation and the negative effects of uremic toxins into the renal system.

## 1. Introduction: Gut Microbiota, Uremic Toxins Definition, and Literature Screening

More and more interest is paid to the prevention and reduction of metabolic and nutrition-associated affections, as the microbial community within the intestine has a fundamental role in the well-functioning of the entire human body [1]. This community is responsible for food matrix degradation, nutrient absorption, substance synthesis, energy provision, and metabolite accumulation and excretion, a fact that makes this ‘organ’ vital for the proper work of most of the physiological systems of the body. That is to say, when the symbiosis between the gut community and the host is disrupted, the risk of developing a wide range of illnesses is increasingly high [2].

There is a rising interest in nutrition and diseases associated with gut microbial transformation [3,4]. Nourishment is consumed by the numerous microbial species that co-exist in the gastrointestinal tract (GIT) and are transformed into different products that have specific roles for the body [5]. There are certain metabolites that exert particular effects that are useful for diverse microbial activities, but some of them accumulate and have a negative impact on certain tissues. Macronutrients such as proteins, lipids, sugars, fibers, and different pharmaceutical products are degraded by the gut microbes from which specific metabolites end up in the renal system triggering diverse kidney-associated affections. Consequently, the substances resulting from specific metabolic processes, but also those of an inorganic nature that circulate within the body fluids which accumulate and prejudice the renal tissue, are known as uremic toxins [6,7]. Uremic toxins may have different natures or sizes but contribute to impaired kidney functions, triggering various renal insufficiencies [8]. Moreover, uremic toxin accumulation affects multiple processes in the body; for example, they activate the immune response and trigger the inflammation process, leading to further increased risk of other physiological affections like atherosclerosis, diabetes mellitus, obesity, or cardiovascular diseases [9,10].

The accumulation of uremic toxins within the renal system may be also attributed to damaged gastrointestinal mucosa by altered microbiota that facilitates an elevated permeability [11]. Disruption of intestinal permeability can lead to the alteration of intercellular junction proteins like claudin-1, occludin, cingulin, and zonula occluden-1. These proteins play a critical role in maintaining the integrity of the intestinal barrier, which regulates the passage of substances, molecules, and ions between the gut lumen and the bloodstream [12,13,14]. In addition, the disruption of tight junction integrity can lead to increased intestinal permeability, commonly referred to as “leaky gut” [15].

Given the progressing nature of statistical data concerning the impact of uremic toxins on human health [16], it is established that a significant proportion of individuals with kidney disorders manifest an aberrant glomerular filtration rate resulting from the build-up of uremic toxins. This occurrence contributes to elevated rates of morbidity and mortality within this patient population [17]. In addition, renal impairment is tightly linked with an altered cardiovascular system as the kidney and heart share the function of maintaining the host’s health homeostasis, so the risk of developing cardiovascular pathologies by people with altered kidney functions is also high [18]. Uremic toxins, which are small molecules that are inadequately eliminated by dialysis therapies, circulate via the bloodstream and gradually accumulate in renal tissues, thereby disrupting their normal functions [10].

The negative effects of the accumulation of uremic toxins have been pointed out through the nineteenth century when the sole practice of evidencing them was through hemodialysis [18]. Since then, these toxic molecules have been the subject of much investigation as their biological activities are directly linked with the development of uremic syndrome [19]. Additionally, uremic syndrome can be defined as the outcome of modifications induced by the storage of a diversity of compounds that could not be discharged by the renal system [20]. According to the European Uremic Toxins Work Group (EUTox) database [21], over 150 uremic solutes have been identified till the present, and the list is increasing over time. The substances that are recognized as uremic toxins are firstly classified into three groups based on their physicochemical properties, as follows: (i) substances with low weight (under 500 Da) which are water-soluble (e.g., urea, creatinine), (ii) substances with medium weight that have around 500 Da (e.g., small peptides, leptins, microglobulins), and (iii) substances with high weight that are mostly protein-bound compounds (including phenols and indoles). Furthermore, uremic toxins are divided by their chemical structure, distribution in the body fluids, and by their molecular mass and volume [18].

**Figure 1:**
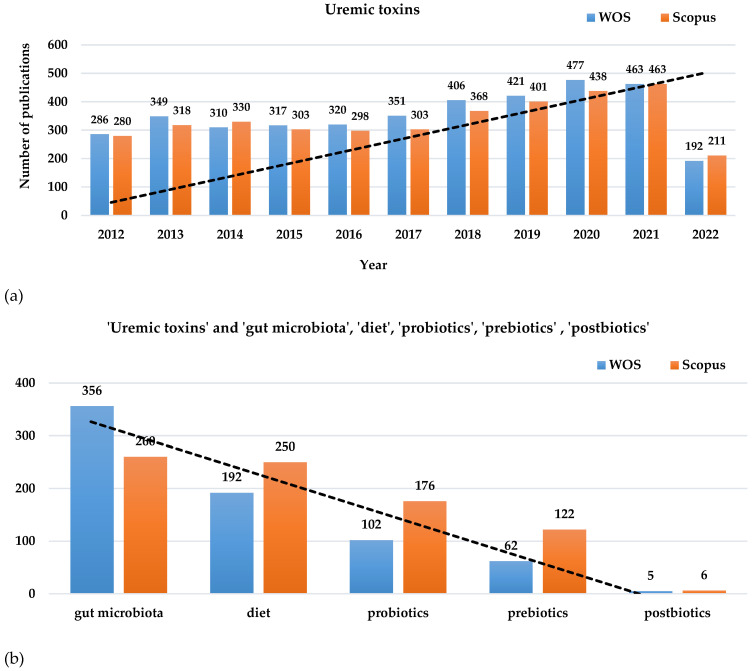
Research papers distributed on Web of Science Core Collection and Scopus in the last decade (2012–2022) related to (**a**) ‘uremic toxins’ and the association with (**b**) ‘gut microbiota’, ‘diet’, ‘probiotics’, ‘prebiotics’, and ‘prebiotics’. The images show an increasing trend line for papers that include ‘uremic toxins’ and a decreasing trend line with the narrow association of uremic toxins with the mentioned terms.

As the prevalence of kidney illnesses based on the implications of uremic toxins has a growing trend [16,22], more attention has been paid to investigating the mechanisms involved in kidney disease progression and in finding non- or minimally invasive alternatives to treatment [23,24]. As diet and lifestyle modulation are among the most investigated non-invasive methods for diminishing kidney-associated affections, multiple scientific publications have published research which has occurred in the last decade on the mentioned topic (Figure 1). Gut microbiota modulation through the use of probiotics and prebiotics for renal illnesses also gained attention, while the impact of probiotics’ metabolites known as postbiotics in close connection with their impact on kidney disease is not sufficiently investigated yet. Based on the scientific search platforms (e.g., Web of Science Core Collection, Scopus), over 3500 publications about ‘uremic toxins’ appeared during the last decade including research articles, clinical trials, review papers, and book chapters during the last decade (Figure 1a). Moreover, among these publications over 250 papers involved associations between uremic toxins and ’gut microbiota’, over 150 papers involved ‘uremic toxins’ and ‘diet’, over 100 papers were associated with ’probiotics’, over 60 papers involved ’prebiotics’, and less than 10 papers involved associations between ‘uremic toxins’ and ’postbiotics’ according to Web of Science Core Collection and Scopus (Figure 1b).

As uremic toxins are actually metabolites generated from different internal processes, they intervene in a series of chronic and non-communicable diseases [25,26]. In the same context, uremic toxins are mainly produced by the microbial community within the gut through the degradation of specific macromolecules, and they further reach the host circulation by influencing other metabolic processes. In addition, if it is properly modulated, the gut community is also able to diminish the formation of metabolites with toxic potential, decreasing the risk of developing associated illnesses by the host at the same time. In this regard, the present manuscript presents the role played by gut microbiota modulation to reduce the accumulation of metabolites that exert toxicity over certain tissues such as renal tissue by the increase in the probiotic community in the GIT. Moreover, the influence of diet and the use of prebiotics for stimulating probiotic growth in association with the reduction of toxins with uremic implications are also considered. Last but not least, the metabolites biosynthesized by the probiotic strains known as postbiotics that exert positive effects over uremic toxicity are covered in the present work.

## 2. Gut Dysbiosis, Uremic Toxicity, and Key Kidney-Associated Diseases

Gut dysbiosis incriminates a significant number of health issues that are reflected afterward in the healthcare system, economy, and society [27]. The intestinal imbalance is also closely connected with the appearance and the progression of kidney diseases and with associated complications such as hypertension, cardiovascular affections, diabetes, and mental or cognitive impairment [14,28,29]. In addition, the gut and renal system intercommunicate through the gut–kidney axis, as the metabolites biosynthesized by the intestinal microbes reach the kidney filtration tissues [30]. Moreover, the microbial community that is found in the colon produces large quantities of metabolic waste that is excreted by the kidneys [22].

The renal system, which is composed of the kidneys, ureters, bladder, and urethra, has the main role of eliminating undesired substances from the body. Supplementarily, kidneys have an additional role in the filtration and recovery of certain compounds [31], a fact that makes them vulnerable to retaining substances with harmful potential [32]. Uremic toxins that can be both water-soluble and liposoluble, have different chemical constructions that play different biological roles, and promote deleterious effects in kidneys [18,33,34]. For instance, in Acute Kidney Injury (AKI), also known as Acute Renal Failure, uremic toxins that are generated from nitrogen metabolism—in particular, urea and creatinine—accumulate and damage the kidney tissue [35,36]. AKI is frequently diagnosed in patients with critical health conditions and appears as an unexpected episode of kidney failure that occurs within a few hours or a few days, and is a contributing factor to short- and long-term complications and elevated mortality rate [31,37]. In AKI, it was observed that the level of creatinine from serum rapidly increases (increases by 0.3 mg/dl or more in 48 h or rises to at least 1.5-fold from baseline within 7 days), while the content of urine is very much reduced. Some of the predominant uremic toxins that appear in AKI are urea, serum creatinine, hippuric acid, indoxyl sulfate, para-cresol sulfate, para-cresol glucuronide, 3-carboxy-4-methyl-5-propyl-2-furanpropionate, indole-3-acetic acid, trimethylamine N-oxide [38]. The complications induced by uremic toxin accumulation in renal tissue degenerate until the diagnosis of Chronic Kidney Disease (CKD). The progression of AKI to CKD is known as “Acute-on-Chronic Kidney Disease” (AoCKD) and occurs when an individual experiences repeated episodes of AKI or if the underlying cause of AKI persists for an extended period, leading to ongoing kidney damage and impaired kidney function over time [39]. CKD represents one of the fastest global causes of mortality, being generally characterized mainly by loss of kidney function. In this particular case, kidney dysfunction is directly proportional to the gut microbiota imbalance, as has been proved by several clinical, in vivo, or in vitro studies [27,40,41,42,43]. Unfortunately, the range of microbiota-derived toxins is increasing, and indoxyl sulfate (which is associated with vascular imbalances) and para-cresyl sulfate (which is responsible for insulin resistance and vascular disease) exemplify the complexity and significance of these compounds in contributing to various health challenges [44,45]. The associated predominant microbiota in AKI and CDK-diagnosed patients was found to be formed of *Escherichia* ssp., *Bacteroidetes* ssp., *Salmonella* ssp., *Clostridium* ssp., *Ruminococcus* ssp., *Rothia* ssp., *Staphylococcus* ssp., *Pseudomonas* ssp., *Streptococcus* ssp., *Enterobacter* ssp., *Faecalibacterium* ssp., and *Lachnospiraceae* ssp. [46,47]. Moreover, most of these pathogenic strains (especially *E. coli*, *P. aeruginosa*, and *S. aureus*) enter the bloodstream and spread to various organs including kidneys causing infections (through invasion and/or toxin elaboration) like urinary tract infections (UTIs), catheter-associated urinary tract infections (CAUTIs), cystitis, pyelonephritis and emphysematous pyelonephritis, glomerulonephritis, tubulointerstitial nephritis, kidney/renal abscesses, or sepsis in critical cases [48,49,50,51,52,53,54].

When the gut–kidney axis is disturbed, the entire body functionality is compromised, including the immune system. In the case of Berger’s disease (an autoimmune disease also known as IgA nephropathy), this is instantiated as an immune renal condition characterized by the deposition of Immunoglobulin A (IgA) antibodies in the glomeruli of the kidneys [55,56]. The exact mechanisms through which the gut microbiota may induce IgA nephropathy are not fully understood, and research in this area is still ongoing. Some hypotheses and associations have been proposed, such as the following: (a) dysbiosis—gut permeability: a compromised gut barrier allows bacterial components, such as lipopolysaccharides, to translocate from the gut into the bloodstream leading to endotoxemia, further triggering an immune response; this immune activation may lead to the production of IgA antibodies against these bacterial components and may contribute to the formation of immune complexes [40,57]; (b) IgA production—immune activation: the gut-associated lymphoid tissue (GALT) is rich in IgA-producing B cells; gut dysbiosis can influence the activation and proliferation of these B cells, leading to the production of more IgA antibodies; some of these IgA antibodies may be directed against gut-derived antigens or bacterial products, and in some instances, these antibodies can cross-react with antigens in the kidneys, contributing to the formation of immune complexes in the glomeruli [58]; (c) genetic factors: genetic predisposition is believed to play a role in the development of IgA nephropathy; certain genetic variations may affect the immune response to gut bacteria and their products, making some individuals more susceptible to the production of autoantibodies and the subsequent development of IgA nephropathy [59]. In IgA nephropathy, it was noticed that gut dysbiosis is induced by an increased presence of bacteria species belonging to *Streptococcus* ssp., *Escherichia*-*Shigella* ssp., and *Eubacterium* ssp., and low contents of *Bifidobacterium* ssp., *Enterococcus* ssp., *Clostridium* ssp., *Leuconostoc* ssp., *Prevotella* ssp., and *Lactobacillus* ssp. were noticed. Moreover, the metabolites produced by the mentioned strains and classified as uremic toxins like ethyl alcohol, 2,6-octadien-1-ol 3,7 dimethyl- (Z), 1-octanol, 4-methyl-phenol, phenol 4-(1,1,3,3-tetramethylbutyl), heptanoic acid 1, 1-dimethyl ethyl ester, hexyl n-valerate, heptanoic acid 1-methyl ethyl ester, benzoic acid hexadecyl ester, and phthalic acid methyl neopentyl ester were found at significantly higher levels in the feces and/or urine of progressive IgA-nephropathy-diagnosed patients [60].

End-stage Renal Disease (ESRD) is the final and most advanced stage of CKD, wherein the kidneys have significantly impaired function, necessitating dialysis as a crucial supportive measure [32]. As a result, the accumulation of uremic toxins in the bloodstream becomes a significant concern. Gut dysbiosis, gut inflammation, and heightened gut permeability are the primary contributing factors leading to the development of ESRD. These factors facilitate the movement of uremic toxins (such as indoxyl sulfate and para cresol sulfate), bacterial components, and pro-inflammatory cytokines from the gut into the bloodstream [17]. In terms of intestinal dysbiosis, in clinical studies conducted on ESRD-diagnosed patients having peritoneal dialysis, hemodialysis, or kidney transplant, significant alterations were observed among commensal microflora, as species belonging to Firmicutes and Actinobacteria were substantially decreased, while species belonging to Bacteroidetes, Proteobacteria, and Enterobacteriaceae (e.g., *E. coli*, *P. aeruginosa*) were largely increased [54,61].

## 3. Lifestyle, Dietary Habits, and Uremic Toxicity

**Figure 2:**
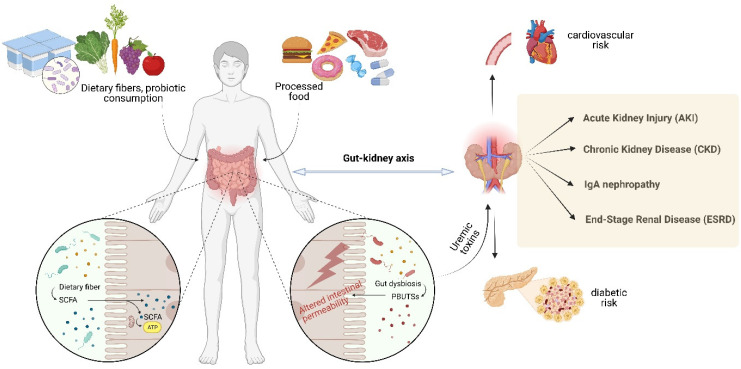
The interconnection between dietary habits, general health state and the development of kidney-associated disorders (Created with BioRender.com; accessed on 22 August 2023).

It is widely known that lifestyle and dietary habits influence one’s general health status and implicitly the development of kidney-associated disorders (Figure 2). In recent studies, diet has been much investigated as the most accessible and non-invasive method of modulating gut microbiota to treat kidney diseases [23,62]. There is a thin line between our diet and the production of uremic toxins, the dietary intake being seen as the main source of substrates for uremic toxin production. Carbohydrates, lipids, proteins, minerals, and micronutrients are the precursors of protein-bound uremic toxins (PBUTSs) [63]. It seems that the everyday foods we eat, either directly or indirectly contribute to the plasma retention of uremic toxins. Furthermore, disturbances in gut microbiota composition, often referred to as dysbiosis, can lead to an imbalance in metabolite production. Dysbiosis may result from factors like dietary habits, medications, physical activity, and underlying health conditions, further impacting kidney function and disease progression [64,65].

Nonetheless, both vegetarian and omnivore diets have their advantages and challenges when it comes to their impact on kidney affections, such as uremia and CKD [66]. A well-balanced vegetarian diet, rich in plant-based nutrients and carefully planned to ensure adequate protein intake, can be beneficial for managing CKD and reducing the risk of uremic toxin formation. On the other hand, an omnivore diet that focuses on lean protein sources, whole grains, fruits, and vegetables while moderating the consumption of red and processed meats can also support kidney health and minimize the risk of uremia-related complications. Individual preferences, nutritional needs, and consultation with healthcare professionals are essential considerations when choosing a dietary approach for individuals with kidney disorders like AKI, CKD, or ESRD [67,68,69].

### 3.1. Carbohydrates

According to their degree of polymerization, carbohydrates are represented by monosaccharides, disaccharides, polyols, oligosaccharides, and polysaccharides. Reducing sugars present in this class (glucose, fructose, galactose, lactose, and maltose) are precursors of some PBUTSs. These compounds intervene in the Maillard reaction mechanism (the reaction between reducing sugars and amino acids that occurs during food treatments at high temperatures). Uremic products obtained from this reaction are fructose-lysine, 3-deoxyglucosone, glyoxal, methylglyoxal, pentosidine, Nε-carboxymethyl lysine, and Nε-carboxyethyl lysine. Fructose-lysine may be found in pasteurized milk, pasta, chocolate, cereals, and carbonated soft drinks, 3-deoxyglucosone is present in honey, and glyoxal and methylglyoxal could be found in an extended range of goods, from bread to cigarettes [70]. Moreover, carbohydrate consumption can lead to the formation of Advanced Glycation End-Products (AGEs), which are also considered uremic toxins. AGEs are a complex group of compounds formed when sugars react with proteins, lipids, or nucleic acids through a process known as glycation [33,71,72]. The accumulation of these small, water-soluble uremic toxins in the bloodstream, when the kidneys are unable to effectively clear them from the body, can contribute to oxidative stress, inflammation, and cellular damage. This, in turn, further worsens kidney function and contributes to the progression of kidney failure. Reducing sugar content in foods is recommended to prevent the generation of uremic toxins. Foods that are rich in sugars, especially when exposed to high temperatures, can also contribute to AGE formation. That is to say, other heat treatments, such as boiling and steam cooking, can be used to achieve this instead of grilling, frying, or roasting. Thus, a diet high in carbohydrates, especially refined sugars and heavily processed foods, can indirectly contribute to the accumulation of AGEs [70]. A diet low in red meat and high in whole grain fibers encourages the proliferation of saccharolytic taxa that lower levels of gut-derived uremic toxin. Furthermore, in kidney-related conditions, close attention is needed regarding carbohydrates and blood sugar control. Monitoring carbohydrate intake is crucial to ensure stable blood sugar levels and prevent complications associated with diabetes, which is a frequent comorbidity in individuals with kidney failure [73].

### 3.2. Lipids

Kidney-related issues can be caused by lipid-based compounds. CKD, especially in its advanced stages, can lead to dyslipidemia, which is an abnormal lipid profile characterized by elevated levels of triglycerides, low-density lipoprotein cholesterol (LDLc), and reduced levels of high-density lipoprotein cholesterol (HDLc). Dyslipidemia is further associated with an increased risk of cardiovascular disease in individuals with CKD [74]. Uremic toxins like indoxyl sulfate and para cresyl sulfate can alter the expression and function of lipid transporters and receptors in the liver and other tissues, leading to dysregulation of lipid metabolism [75]. For example, indoxyl sulfate has been shown to disrupt cholesterol homeostasis and promote lipid accumulation in macrophages, potentially contributing to atherosclerosis and cardiovascular complications. Furthermore, uremic toxins induce oxidative stress, which, in turn, leads to dyslipidemia by altering lipoproteins, increasing their atherogenicity, and contributing to the advancement of atherosclerosis [76]. Nonetheless, increased levels of uremic toxins may contribute to lipotoxicity, a condition where excessive lipids in tissues, such as the kidney and blood vessels, lead to cell dysfunction and damage [77]. Regardless of this, the intake of dietary fats can influence the accumulation and clearance of uremic toxins in individuals with kidney disease. The composition of dietary lipids, particularly the types of fats consumed, can impact the production of uremic toxins in the body. For example, diets high in unhealthy fats, such as saturated and trans fats found in processed foods and fatty meats, contribute to oxidative stress and inflammation, leading to the generation of uremic toxins [33]. When oxidative stress is present in the body, lipids, especially LDLc, can undergo oxidation. This process leads to the formation of uremic toxins derived from lipids, such as lipid hydroperoxides and oxysterols. These substances can exacerbate kidney injury and contribute to its progression [33,78]. Last but not least, adequate lipid intake, especially essential fatty acids like omega-3 polyunsaturated fatty acids found in fish and plant-based sources, may positively influence the clearance of uremic toxins. Studies have suggested that omega-3 fatty acids can improve kidney function and reduce inflammation, potentially aiding in the removal of uremic toxins [71].

### 3.3. Proteins

Proteins are directly implicated in the formation of uremic toxins, and their accumulation can play a significant role in kidney failure and the progression of AKI, CKD, and ESRD. As proteins are essential macronutrients that undergo metabolism in the body to provide amino acids, the building blocks necessary for various physiological processes, waste products and by-products are generated, including urea and creatinine, guanidino compounds like guanidino acetic acid, guanidino succinic acid, and guanidino butyric acid, indoxyl sulfate, para cresyl sulfate, indole-3-acetic acid, and homocysteine [79]. These waste products are typically filtered out of the blood by healthy kidneys and excreted in the urine [80,81,82]. Uremic toxins derived from protein metabolism can lead to oxidative stress, inflammation, and cellular damage in the kidneys [68,83]. The amount and quality of dietary protein intake can also influence protein-derived uremic toxin formation. Diets that are high in protein content, especially animal protein, may increase the production of specific uremic toxins. Therefore, in individuals with kidney disease, dietary protein intake may need to be moderated or adjusted based on the severity of kidney impairment [84]. In addition, in cases of advanced kidney failure, there may be an increased risk of protein-energy wasting (PEW), where the body breaks down muscle protein for energy due to inadequate nutrient intake and chronic inflammation. PEW can lead to malnutrition and further contribute to the accumulation of uremic toxins [85,86]. It was observed that anuric hemodialysis patients with a low protein/fiber index exhibited lower levels of uremic toxins like indoxyl sulfate and para cresyl sulfate in their blood. The blood concentrations of these uremic toxins were strongly influenced by the patient’s diet, showing a significant correlation with the protein/fiber index. That is to say, to investigate the potential reduction of these uremic toxins, diets with higher fiber intake should be tested as a beneficial approach to lowering their levels and minimizing the risk of undernutrition [87].

### 3.4. Minerals

Minerals also play a significant role in the formation and clearance of uremic toxins in the context of kidney failure and kidney diseases. Minerals like phosphate, calcium, magnesium, potassium, sodium, or trace minerals may influence both the progression of the disease and the overall health of individuals with impaired kidney function [88]. For instance, in kidney failure, the kidneys lose their ability to effectively excrete phosphate, leading to elevated levels of serum phosphate (hyperphosphatemia). High phosphate levels can bind with calcium, causing a decrease in serum-ionized calcium levels. In response, the body may release parathyroid hormone (PTH) to maintain calcium levels. In addition, prolonged elevation of PTH leads to secondary hyperparathyroidism, contributing to bone disease and vascular calcification [89,90]. CKD-diagnosed patients use phosphate binder medication to manage elevated phosphate levels. These binders work by reducing the absorption of dietary phosphate from the gastrointestinal tract, thereby helping to control phosphate levels in the blood. Phosphate binders, when taken with meals, work by binding to dietary phosphate in the gastrointestinal tract [91]. This binding reduces the amount of phosphate available for absorption into the bloodstream. There are different types of phosphate binders, including calcium-based, aluminum-based, magnesium-based, and non-calcium-, non-aluminum-based binders [91,92].

The kidneys are also responsible for maintaining a proper balance of magnesium and potassium in the blood. In kidney failure, reduced kidney function can lead to the accumulation of magnesium (hypermagnesemia) and potassium (hyperkalemia) in the bloodstream. These imbalances lead to adverse effects on the heart and nerve function, triggering cardiovascular complications [93]. On the other hand, sodium intake is usually restricted in individuals with kidney failure to manage fluid retention and blood pressure. Excessive sodium intake leads to fluid retention and edema, putting additional strain on the kidneys and exacerbating kidney damage [94,95]. Certain trace minerals, such as zinc and selenium, are essential components of antioxidant defense systems, and in kidney failure cases, imbalances in trace minerals can contribute to oxidative stress, inflammation, and cellular damage, further impairing kidney function, and overall health [96]. In addition, kidney failure can lead to anemia due to reduced production of erythropoietin, a hormone that stimulates red blood cell production. Iron deficiency is a common cause of anemia in individuals with kidney failure, so iron supplements or intravenous iron therapy may be prescribed to manage anemia in these patients [97]. Nonetheless, kidneys play a vital role in maintaining acid–base balance in the body. When renal function is compromised, acidosis (increased acidity) may develop due to reduced acid excretion. Acidosis can lead to metabolic disturbances and further contribute to kidney injury [98].

### 3.5. Bioactive Compounds

Bioactive compounds are naturally occurring substances found in certain foods that have specific physiological effects on the human body [99]. Some studies have suggested that certain bioactive compounds, like polyphenols found in fruits, vegetables, green tea, or turmeric, may have protective effects on kidney function [3]. These polyphenols have been shown by various studies to support kidney health and mitigate the progression of kidney injury by modulating different signaling pathways and inflammatory responses. However, while polyphenols are generally praised for their ability to neutralize harmful free radicals and reduce oxidative stress, it is important to note that some of these compounds can become detrimental when they accumulate in individuals with kidney failure [100]. Some researchers consider polyphenols as PBUTS precursors [70,101]. For instance, hippurates, which are considered uremic toxins, can be formed not only through the conversion of chlorogenic acid and (+)-catechin but also from various other phenolic compounds, such as caffeic acid, flavan-3-ol, cyanidin, and quercetin [70]. A recent cross-sectional study in Australia did not find any association between the intake of fruits and vegetables, which are the main sources of polyphenols, and plasma levels of para cresyl sulfate in hemodialysis patients. While more research is needed to establish a direct link between polyphenol intake and uremic toxicity, considering their protective role as antioxidants, it is recommended not to exclude polyphenols from the diet in CKD [70,102].

### 3.6. Vitamins

In individuals with kidney failure, especially those undergoing dialysis, there is an increased risk of vitamin deficiencies due to reduced dietary intake, impaired absorption, and loss during dialysis [103]. Common deficiencies may include water-soluble vitamins like B-complex vitamins (B1, B6, B12, folic acid) and fat-soluble vitamins (A, D, E, K). In the case of vitamin C (ascorbic acid), which is a water-soluble and strong antioxidant vitamin, can help neutralize free radicals and reduce oxidative stress, but in advanced kidney failure, the accumulation of oxalic acid which is a byproduct of vitamin C metabolism can contribute to the formation of kidney stones [104]. Deficiencies of B-complex vitamins, such as thiamine (B1), pyridoxine (B6), and cyanocobalamin (B12), can lead to neurological complications, including peripheral neuropathy and encephalopathy, commonly observed in individuals with advanced kidney failure [105]. Folic acid deficiency can exacerbate anemia in individuals with kidney failure, as it plays a vital role in red blood cell formation [106]. Vitamin D plays a crucial role in calcium and phosphorus metabolism and bone health. In kidney affections, impaired kidney function can lead to a decrease in the active form of vitamin D (calcitriol) production, leading to disturbances in calcium and phosphorus levels, bone disease, and secondary hyperparathyroidism [107]. In kidney diseases, disturbances in vitamin A metabolism can also occur, leading to higher levels of retinol-binding protein and alterations in vitamin A storage and transport [108].

Overall, maintaining a balanced diet that includes a variety of foods rich in macronutrients, vitamins, minerals, and antioxidants is crucial for supporting kidney health and managing uremic toxins in individuals with kidney failure. It is essential to work closely with healthcare professionals, including registered dietitians and nephrologists, to develop a personalized dietary plan that meets individual nutritional needs and helps improve overall well-being in kidney disease patients.

## 4. Biotics (Pre-, Pro-, Post-) and Uremic Toxicity: Prevention and Possible Therapy

**Figure 3:**
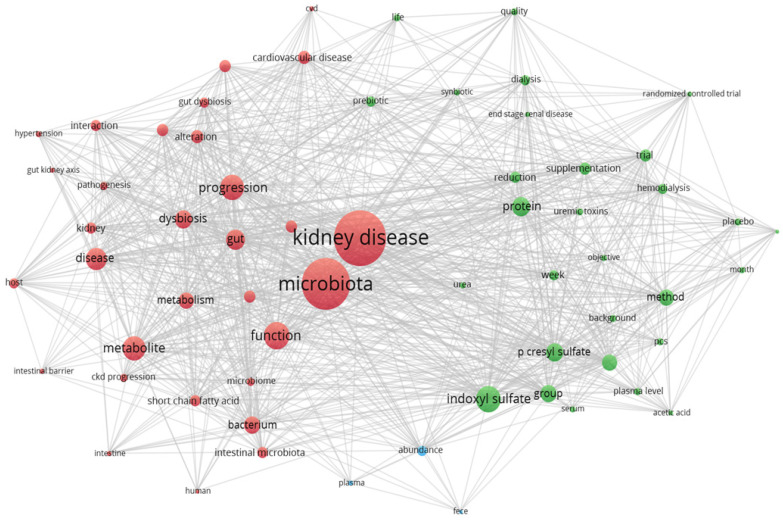
The gut, microbiota, metabolites, and kidney-related conditions are closely linked and interdependent. This intricate interplay between the gut and the kidneys has a profound impact on kidney health and can significantly affect the development and progression of kidney-related conditions (VOSviewer version 1.6.17).

The relationship between the gut, microbiota, major and/or secondary metabolites, and kidney-related conditions is highly interconnected and interdependent. This intricate network of interactions influences kidney health and can have significant implications for kidney-related conditions [109]. The interplay between the gut, microbiota, metabolites, and the kidneys is part of a bidirectional relationship known as the gut–kidney axis. Changes in the gut microbiota can influence kidney function, and kidney dysfunction can, in turn, alter the gut microbiota composition [110]. Understanding and manipulating this intricate relationship holds promise for developing new therapeutic approaches for kidney-associated affections. The strong interconnection between the gut and kidneys and other related aspects can be seen in Figure 3.

### 4.1. Prebiotics

As a general description, prebiotics are non-digestible fibers that serve as food for beneficial bacteria in the gut [14]. The quality of prebiotics can positively influence the composition and diversity of the gut microbiota. A healthy and balanced gut microbiota is crucial for the metabolism and clearance of uremic toxins; thus, beneficial gut bacteria can help reduce the production of certain uremic toxins like indoxyl sulfate and para cresyl sulfate that are produced by harmful bacteria like *Clostridium* or Enterobacteriaceae by metabolizing precursors in the gut [111]. Prebiotic substrates such as inulin, fructo-oligosaccharides, and β-glucans enhance the gut barrier function, reducing the translocation of bacterial components and pro-inflammatory cytokines from the gut into the bloodstream. Prebiotics stand at the basis for forming postbiotics, i.e., the extremely valuable SCFAs acetate, propionate, and butyrate. These help mitigate inflammation and oxidative stress, which are contributing factors to most kidney injuries [24,41,112,113]. Nonetheless, prebiotic substrates positively impact the gut–kidney axis, leading to improved kidney health and the clearance of uremic toxins [27,114]. Furthermore, several studies have provided evidence supporting the use of prebiotics as a nutritional therapy for managing kidney-related disorders [24,111,115]. The manipulation of the gut microbiota using prebiotics and other dietary interventions is an area of ongoing research. Modulating the gut microbiota with prebiotics and promoting the growth of beneficial bacteria may have therapeutic potential in managing kidney disorders and reducing the burden of uremic toxins.

### 4.2. Probiotics

Beneficial bacteria commonly recognized as probiotics can reduce the formation and accumulation of uremic toxins like indole, phenol, or para cresol by metabolizing their precursors like phenylalanine, tyrosine and tryptophan, which are amino acids found in the diet [110,116]. The probiotic strains keep the integrity of the intestinal barrier by preventing the leakage of harmful substances like uremic toxins from the gut into the bloodstream. In addition, probiotics like *Bifidobacteria* and lactobacilli were proven to have the ability to restore damaged colonic epithelial tight junctions and to stimulate the secretion of intestinal mucus, leading to a reduction in systemic inflammation at the same time—a process closely linked to uremic toxin accumulation and kidney-related conditions [10,117,118]. Multiple in vivo, in vitro, or clinical studies (e.g., Table 1 and Table 2) argue for the beneficial effect of probiotic strains in diminishing the symptoms and damage of kidney injuries. For example, a particular clinical study performed by Eidi and their team (2018) on 42 hemodialysis patients (32 male and 10 female) proved the efficacy of a probiotic treatment in patients undergoing hemodialysis. In this case, the probiotic strain *L. rhamnosus* administered for 4 weeks provided lower values for uremic toxins (p-cresol and phenol) compared with the placebo group [43]. Furthermore, recent meta-analyses focusing on the effects of biotic supplements in individuals with kidney conditions, particularly CKD and ESRD, provide robust evidence of their advantageous impact. These studies strongly endorse their ability to decrease levels of serum uremic toxins such as indoxyl sulfate, p-cresyl sulfate, blood urea nitrogen, malondialdehyde, and serum creatinine. Additionally, these supplements have shown promise in alleviating gastrointestinal symptoms and enhancing the quality of life among CKD and ESRD patients [24,119]. To sum up the main scientific outcome in this context, probiotics create an environment that is less favorable for uremic toxin accumulation.

### 4.3. Postbiotics

Among the essential metabolites produced by probiotic bacteria when they grow on non-fermentable fibers are bioactive compounds like peptides, organic acids, and short-chain fatty acids (SCFAs), collectively known as postbiotics [120,121]. Notably, SCFAs, such as acetate, propionate, and butyrate, have demonstrated the ability to reduce oxidation and inflammatory processes, consequently lowering the accumulation of uremic toxins in both the bloodstream and renal tissue [122]. Moreover, a six-month study conducted on senior adult cats revealed promising outcomes when they were fed a control maintenance diet supplemented with various nutrients [109]. The supplemented diet exhibited several positive effects, including increased lean-body percentage, maintained serum albumin concentrations, higher glomerular filtration rate (GFR), and decreased serum symmetric dimethylarginine (SDMA) concentrations. Additionally, the cats displayed lower plasma metabolite concentrations, indicative of reduced oxidative stress and a shift in methyl group allocation. Furthermore, the consumption of the supplemented food resulted in changes in microbial postbiotics, leading to a decrease in the plasma concentration of the uremic toxin 3-indoxyl sulfate. These observed changes have the potential to counteract the sarcopenia and chronic inflammation typically associated with aging in cats. However, further research is required to validate these health benefits and ascertain the anti-aging effects in senior adult cats [109]. Nevertheless, the use of postbiotics, particularly SCFAs, holds promise as a novel therapeutic approach for mitigating the effects of uremic toxins and supporting kidney health. Additionally, these findings suggest that tailored dietary interventions, including postbiotics and nutrient supplementation, may play a pivotal role in improving kidney function and overall health outcomes in both human and animal populations. Still, more extensive investigations are essential to fully comprehend the precise mechanisms and long-term effects of postbiotics in uremic toxin management and age-related kidney conditions.

**Table 1:** Examples of major kidney-associated affections triggered by uremic toxins and pre-/pro/postbiotic treatments (pre-clinical data).

Disease	Identified Uremic Toxins	Type of Study	Biotic Treatment (Pre-/Pro-/Post-)	Main Study’s Results	Ref.
AKI	Indoxyl sulfate, p-cresol, phenol	In vivo—on male rats with cisplatin-induced AKI; In vitro—cultivated in MRS broth with indoxyl sulfate, p-cresol, and phenol for 24 and 48 h	Probiotics: *Lactobacillus plantarum* BCRC12251, *L*. *paracasei* BCRC12188, *Streptococcus salivarius* subsp. *thermophilus* BCRC13869	Suppressed the accumulation of IS in the serum; reduced the level of IS after 48 h	[123]
	Indoxyl sulfate, p-cresol sulfate	In vivo—on rats with cisplatin-induced AKI; In vitro—on Caco-2 cell line	Probiotic: *Lactobacillus* *salivarius* BP121	Reduced renal inflammation and oxidative stress; reduced intestinal permeability	[124]
CKD	Indoxyl sulfate, p-cresol sulfate, phenyl-acetyl-glutamine, and trimethylamine N-oxide	In vivo—on adenine-induced CKD in rodent models	Probiotic: *Bifidobacterium animalis* A6	Decreased abundance of *Eggerthella lenta* and *Fusobacterium nucleatum* and reduced levels of toxins and the severity of the disease	[42]
	Hippuric acid, 3-carboxy-4-methyl-5-propyl-2-furan propionate, indole-3-acetic acid, indoxyl sulfate, p-cresol sulfate, para-cresyl glucuronide, trimethylamine N-oxide	In vivo—on adenine-induced CKD in rats	Probiotics: *Bacillus subtilis* TO-A, *Enterococcus faecium* T-110, and *Clostridium butyricum* TO-A	Decrease in intestinal pH by increasing SCFA production that further suppressed the production of uremic toxins	[125]
	Indole, p-cresol, p-cresyl sulfate	In vitro—on fecal batches collected from healthy and CKD subjects	Probiotic: *Bifidobacterium animalis* BLC1, *Lacticaseibacillus casei* LC4P1; Prebiotic: inulin, fructooligosaccharides, quercetin, resveratrol, and proanthocyanidins	On fecal batches collected from CKD—modified the viable cell densities of some cultivable bacterial patterns, and increased the concentration of acetic acid and decane, while reducing the concentration of nonanoic acid, dimethyl trisulfide, and indoxyl sulfate	[41]
IgA neprop-athy		In vivo—on IGA-induced mouse model	Probiotic: *Bifidobacterium longum* and *Lactobacillus bulgaricus*	Alleviation in gut dysbiosis associated with induction of IgA nephropathy	[126]

**Table 2:** Examples of major kidney-associated affections triggered by uremic toxins and pre-/pro/postbiotic treatments (clinical data).

Disease	Identified Uremic Toxins	Type of Study	Biotic Treatment (Pre-/Pro-/Post-)	Main Study’s Results	Ref.
CKD	Indoxyl sulfate, p-cresol sulfate	In vivo study—on stage 3–4 CKD patients; 6-week, double-blind, placebo-controlled, parallel-arm, randomized controlled trial	Prebiotic: high-amylose maize-resistant starch type 2 (RS-2)	Reduction in indoxyl sulfate and p-cresol sulfate, reduction in key markers of inflammation	[111]
	Indoxyl sulfate, p-cresyl sulfate	In vivo study—on 37 predialysis adult participants with CKD; 6 weeks, randomized, double-blind, placebo-controlled, crossover trial	Synbiotic therapy; prebiotic: high-molecular-weight inulin (inulin high-performance), fructo-oligosaccharides, and galacto-oligosaccharides (GOSs); probiotic: nine different strains across the *Lactobacillus*, *Bifidobacteria*, and *Streptococcus* genera	A low reduction of serum indoxyl sulfate, and a significant reduction of serum p-cresyl sulfate; alteration in stool microbiome, particularly with enrichment of *Bifidobacterium* and depletion of *Ruminococcaceae*	[127]
	p-cresyl sulfate, p-cresyl glucuronide, indoxyl sulfate, trimethy- lamine N-oxide, phenylacetylglutamine	In vivo study—on 40 participants with CKD not yet on dialysis; 4 weeks, randomized, placebo-controlled, double-blind, cross-over study design.	Prebiotic: arabinoxylan oligosaccharides (AXOS) and maltodextrin	No significant effect of AXOS on serum p-cresyl sulfate, p-cre- syl glucuronide, indoxyl sulfate, and phenylacetylglutamine. A small, albeit significant, decreasing effect on serum trimethylamine N-oxide was observed. No effect of AXOS on 24 h urinary excretion of p-cresyl sulfate, p-cresyl glucuronide, indoxyl sulfate, and phenylacetylglutamine, nor on trimethylamine N-oxide	[128]
	p-cresol	In vivo study—on 13 individuals with CKD; 12-week, single-blind study	Prebiotic: sucrose, pea hull fiber, inulin	Plasma p-cresol decreased from 7.25 ± 1.74 mg/L during week 1 to 5.82 ± 1.72 mg/L during week 12	[129]
IgA nephropathy		Clinical study—on 35 IgA nephropathy-diagnosed patients and 25 healthy controls; 8 weeks, randomized controlled trial	Probiotic: *Bifidobacterium longum* and *Lactobacillus bulgaricus*	Both probiotics and their SCFA metabolites could attenuate the clinicopathological manifestations of IgAN by inhibiting the NLRP3/ASC/Caspase 1 signaling pathway	[126]
ESRD	Urea, hippuric acid, phenyl-acetyl-glutamine	Clinical study—on a cohort of 12 ESRD patients and 12 healthy controls; randomized controlled trial	Probiotic: *Bifidobacterium animalis* A6	Decreased abundance of *Eggerthella lenta* and *Fusobacterium nucleatum* and reduced levels of toxins and the severity of the disease	[42]
	Indoxyl sulfate and para cresol sulfate	Pilot study—on 20 patients on maintenance hemodialysis on systemic inflammation; 12 weeks, single-center non-randomized pilot study	Postbiotic: sodium propionate (SP)	Reduction in pro-inflammatory parameters and oxidative stress and improved insulin resistance and iron metabolism. SP effectively lowered uremic toxins indoxyl and para cresol sulfate	[130]

Research in this field is ongoing, and ongoing studies continue to shed light on the complex interactions between the gut, microbiota, metabolites, and kidney health. As the understanding of this interconnection deepens, targeted interventions involving dietary modifications, prebiotics, probiotics, and other treatments may emerge to support kidney health and manage kidney-related conditions more effectively. However, it is important to acknowledge the limitations inherent in this line of investigation. Studies often exhibit heterogeneity in design, sample sizes, and outcomes, making direct comparisons challenging. Small sample sizes, short follow-up periods, and variability in pre-/pro-/postbiotic compositions can hinder the generalizability of findings. Additionally, the lack of standardized protocols and understanding of mechanisms, along with potential confounding factors and publication bias, further complicate the interpretation of results. Nonetheless, a holistic approach that addresses the gut kidney axis may pave the way for improved outcomes and better management of kidney-associated affections.

## 5. Conclusions and Future Perspectives

In conclusion, the emerging field of research on the interplay between gut microbiota, dietary habits, metabolites, uremic toxins, and kidney disorders has revealed significant insights into potential therapeutic strategies for managing kidney health and related conditions. The gut microbiota, consisting of a diverse community of microorganisms, plays a crucial role in the metabolism of dietary components and endogenous compounds. This intricate network of interactions influences kidney health and can have profound implications for the accumulation and elimination of uremic toxins, which are associated with kidney dysfunction. The consumption of a balanced diet, rich in prebiotics, probiotics, and dietary fibers, has been shown to promote the growth of beneficial gut bacteria and foster the production of beneficial metabolites known as postbiotics like SCFAs. SCFAs, such as acetate, propionate, and butyrate, exhibit anti-inflammatory and antioxidant properties that can indirectly reduce uremic toxin formation and accumulation. However, the specific effects of these interventions may vary among individuals due to differences in gut microbiota composition, diet, and health status. Personalized approaches to diet and supplementation, guided by healthcare professionals, are crucial for optimizing the benefits of these interventions in managing kidney disorders. Future perspectives in this area of research are promising. As technology and knowledge advance, further studies can explore the intricate mechanisms underlying the gut–kidney axis and the impact of dietary habits on gut microbiota. Unraveling the specific interactions between gut metabolites, uremic toxins, and kidney function will pave the way for more targeted therapies and nutritional interventions. Furthermore, translational research is essential to bring these findings from preclinical and animal models to clinical trials in human populations. Long-term studies evaluating the effects of dietary modifications, gut microbiota modulation, and postbiotic supplementation in individuals with kidney disorders will provide valuable data for evidence-based therapeutic recommendations.

In conclusion, the relationship between gut microbiota, dietary habits, metabolites, uremic toxins, and kidney disorders is a multifaceted and dynamic area of research. By harnessing the power of the gut microbiota and its metabolites through targeted dietary approaches, we may be able to make strides in preventing, managing, and potentially even reversing kidney-related conditions, ultimately improving the quality of life for affected individuals.

## Data Availability

Not applicable.
